# Target Recognition for Ultra-Wideband Radio Fuzes Using 1D-CGAN-Augmented 1D-CNN

**DOI:** 10.3390/e27090980

**Published:** 2025-09-19

**Authors:** Kaiwei Wu, Shijun Hao, Yanbin Liang, Bing Yang, Zhonghua Huang

**Affiliations:** School of Mechatronical Engineering, Beijing Institute of Technology, Beijing 100081, China; wukaiwei@bit.edu.cn (K.W.); 3120215165@bit.edu.cn (S.H.); liangyanbin@bit.edu.cn (Y.L.); 3120215164@bit.edu.cn (B.Y.)

**Keywords:** ultra-wideband, fuze, target recognition

## Abstract

In ultra-wideband (UWB) radio fuzes, the signal processing unit’s capability to rapidly and accurately extract target characteristics under battlefield conditions directly determines detonation precision and reliability. Escalating electronic warfare creates complex electromagnetic environments that compromise UWB fuze reliability through false alarms and missed detections. This study pioneers a novel signal processing architecture. The framework integrates: (1) fixed-parameter Least Mean Squares (LMS) front-end filtering for interference suppression; (2) One-Dimensional Convnlutional Neural Network (1D-CNN) recognition trained on One-Dimensional Conditional Generative Adversarial Network (1D-CGAN)-augmented datasets. Validated on test samples, the system achieves 0% false alarm/miss detection rates and 97.66% segment recognition accuracy—representing a 5.32% improvement over the baseline 1D-CNN model trained solely on original data. This breakthrough resolves energy-threshold detection’s critical vulnerability to deliberate jamming while establishing a new technical framework for UWB fuze operation in contested spectra.

## 1. Introduction

A radio fuze is a device that controls the detonation of ammunition by utilizing environmental or target information according to predetermined strategies [1]. As the ‘final link’ in the informationization of weapon systems, the fuze occupies an irreplaceable position in weapon systems, and the effectiveness of radio fuzes directly determines whether the ammunition system can achieve maximum destructive effects [2,3]. The UWB fuze is a specialized type of radio fuze that detects targets by transmitting nanosecond-level ultra-narrow pulses, offering advantages such as high resolution and superior radio frequency stealth performance [4]. Due to its extremely low power spectral density, it can evade detection by enemy fuze jammers, thereby reducing the risk of being jammed, and demonstrates significant advantages in electronic warfare within informationized battlefield environments [5].

However, the noise superimposed on the fuze echo signal in complex electromagnetic environments, combined with ground clutter interference, results in fuze burst height dispersion [6], challenging the target recognition accuracy of UWB fuzes. Meanwhile, advancements in RF hardware circuits and signal processing technologies have improved both the reception sensitivity and jamming capabilities of fuze jammers [7,8,9,10], thereby compromising UWB fuze reliability. In particular, for UWB fuzes, traditional echo signal processing methods—typically based on time-domain energy detection—excessive reliance on signal amplitude makes the fuze susceptible to premature detonation due to noise and interfering signals [11]. Therefore, for UWB fuzes, research on signal processing and target detection methods is imperative to adapt to modern complex battlefield environments.

Beyond conventional energy-threshold detection, target detection methods for UWB fuzes converge on two dominant approaches in emerging research: The first approach utilizes signal transformation techniques (e.g., wavelet transform, singular spectrum analysis, time-frequency analysis) to extract discriminative features via domain conversion [12,13,14], but suffers from high computational complexity and poor real-time performance. The second approach utilizes deep learning architectures to autonomously acquire intrinsic signal patterns for target recognition, removing dependence on amplitude-based features. While research specific to UWB fuzes remains limited, the proven efficacy of deep learning in other fuze systems [15,16] and in broader UWB signal processing domains establishes its technical viability as a promising solution [17,18,19,20,21]. However, its high data dependency results in suboptimal performance in small-sample scenarios. Collecting substantial fuze echo signals demands significant resource investment, inherently limiting dataset scale. This scarcity risks neural network overfitting and degraded target recognition accuracy.

To overcome these challenges, this study proposes a novel signal processing unit architecture featuring: (1) offline-trained LMS adaptive filtering for front-end noise suppression; (2) 1D-CGAN-based augmentation of raw echo signals; and (3) 1D-CNN target recognition trained on the enhanced dataset. Achieving a statistically significant 5.32% increase in recognition accuracy over the non-augmented 1D-CNN baseline, with 0% false alarm and miss detection rates in test scenarios, while enabling real-time processing through 512-point sliding windows with 50% frame overlap. The architecture fundamentally resolves the core limitations of conventional energy-threshold detection methods in contested electromagnetic environments.

## 2. Methodology

### 2.1. UWB Fuze

**Figure 1:**
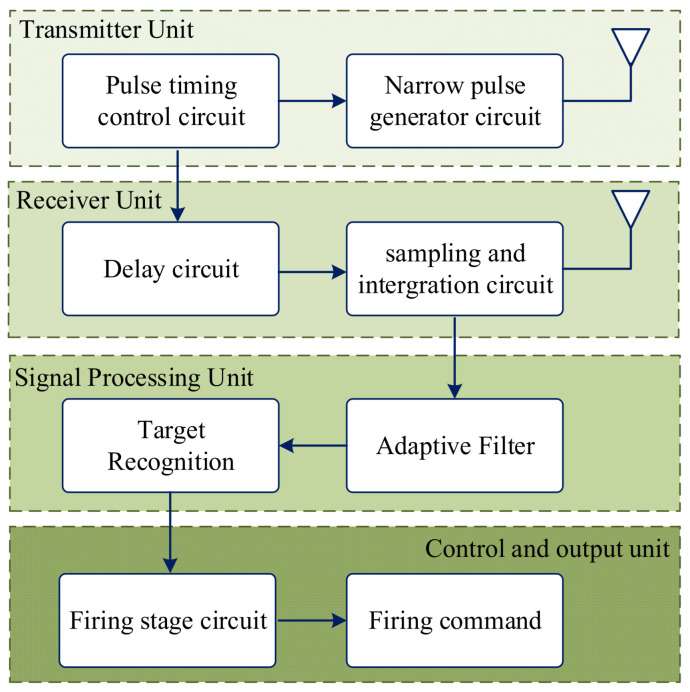
Operation of UWB radio fuze.

The principle block diagram of the UWB fuze ranging system is shown in Figure 1. It is primarily composed of a transmitter unit, a receiver unit, a signal processing unit, and a control and output unit. The transmitter unit comprises a pulse oscillating circuit and a narrow pulse generation circuit. The trigger signal generated by the pulse oscillating circuit controls the narrow pulse generation circuit to produce nanosecond-level pulse signals, which are then radiated through the transmitting antenna. The receiver unit consists of a delay circuit and a sampling pulse generation circuit. The trigger signal from the transmitting unit’s pulse oscillating circuit undergoes a predetermined delay via the delay circuit, subsequently triggering the sampling pulse generation circuit to generate a sampling pulse that functions as a range gate. When the distance between the munition and the ground target matches the preset distance configured in the delay circuit, the sampling pulse circuit samples the echo signals received by the antenna and transmits the target echo signals to the signal processing unit. The signal processing unit conducts filtering and target recognition processing. If the signal characteristics satisfy the target identification criteria, it commands the firing circuit to output an ignition signal, initiating fuze detonation.

The signal processing unit must rapidly and precisely extract target characteristics from raw signals, especially in noise-cluttered environments or deliberate jamming scenarios. This capability enables reliable ignition command triggering through the control unit, which is critical for ensuring robust anti-jamming capability and precise target identification in contested electromagnetic environments.

**Figure 2:**
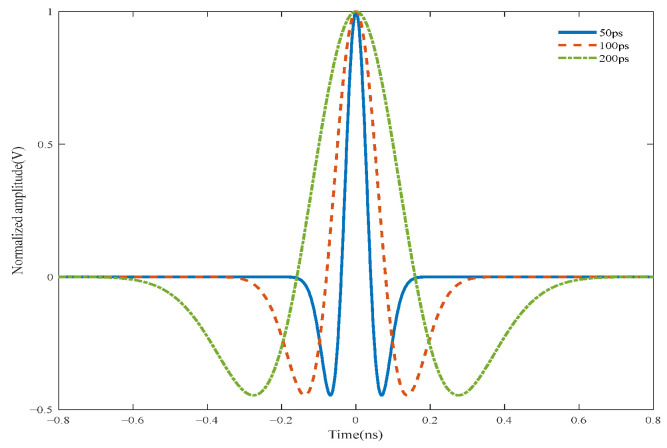
Time-domain waveforms of UWB fuze transmission signals.

In this study, the transmitter signal s(t) is defined as the second derivative of the Gaussian function as follows:

(1) $$s(t)=\left(1-\frac{2\pi t^{2}}{\Delta T^{2}}\right)\exp\left[-\pi (\frac{t}{\Delta T}{)}^{2}\right],$$

where Δ*T* is the pulse width parameter. Figure 2 presents the time-domain waveforms of UWB fuze transmission signals under different pulse width conditions.

The narrow pulse radiates through the transmitting antenna into space, undergoes propagation delay and attenuation to reach the ground, and the ground-reflected signal undergoes propagation delay and attenuation again to arrive at the receiving antenna. Due to the effects of path loss, antenna gain, and radar cross section (RCS), the intensity of the transmitted signal attenuates with increasing distance. Additionally, time delays resulting from varying distances between the fuze and the target scattering centers are manifested in the received signal. The received signal can be expressed as:

(2) $$r(t)=\sum_{m=1}^{P}a_{m}s(t-\tau_{m})+n_{0}(t),$$

where P is the number of scattering centers, s(t) and $n_{0}(t)$ respectively represent the transmitted Gaussian pulse signal and transmission path noise, $a_{m}$ and $\tau_{m}$ are the amplitude attenuation and time delay caused by the m-th scattering point.

### 2.2. Experimental Echo Signal Acquisition

**Figure 3:**
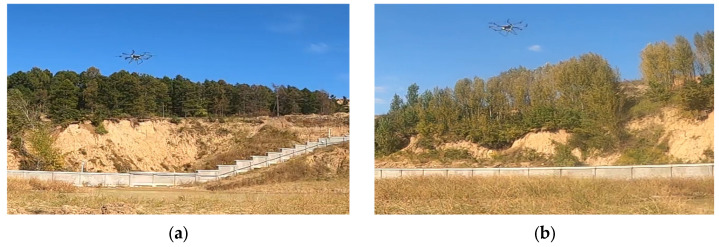
Field data acquisition scenarios. (**a**) Water surface; (**b**) grass surface.

To acquire UWB fuze echo signals for designing and validating signal processing modules, an UWB fuze prototype system was integrated onto a multi-rotor UAV platform for field data collection. During experiments, the UAV executed vertical descent maneuvers over ground targets, simulating terminal engagement scenarios at low airspeeds. The data acquisition system recorded 40 sets of receiver output signals at a sampling frequency of 25 kHz. The experimental configuration is illustrated in Figure 3.

**Figure 4:**
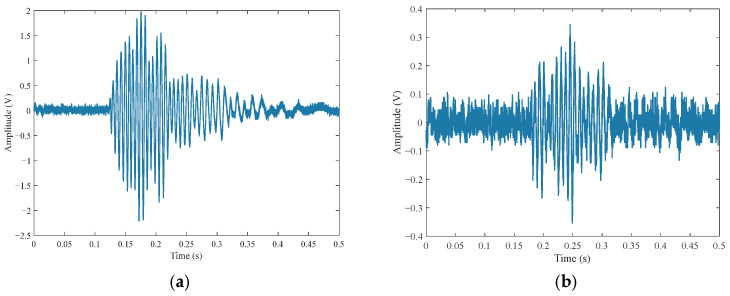
Experimentally collected original echo signals. (**a**) Water surface; (**b**) grass surface.

As shown in Figure 4, the experimentally collected original echo signals exhibit background noise and result from coherent superposition of multiple distinct pulses. Notably, water surface echoes demonstrate larger effective signal peaks with lower noise levels, whereas grass surface echoes exhibit smaller signal peaks and higher noise contamination.

### 2.3. GAN-Based Synthetic Echo Signal Generation

Deep learning-based target recognition methods require substantial training data. Insufficient samples often lead to overfitting, compromising recognition accuracy. However, acquiring large volumes of UWB fuze echo signals demands specific environmental conditions, specialized equipment, and significant human and temporal resources. To address this, GANs can learn the underlying distribution of real samples to generate highly realistic synthetic data, thereby enriching training datasets and enhancing classifier robustness [22,23,24].

**Figure 5:**
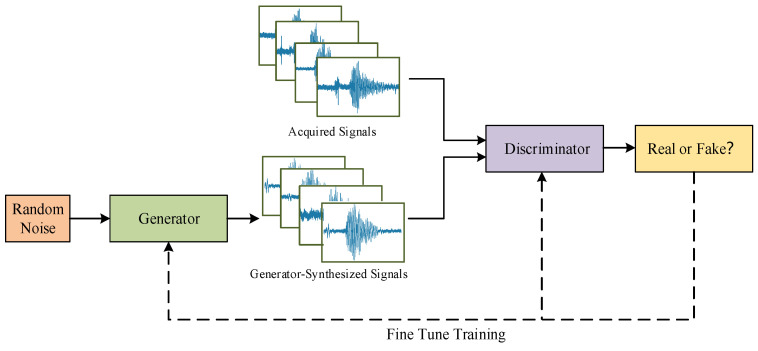
Framework of the 1D-CGAN Network.

Figure 5 illustrates the framework of 1D-CGAN, comprising a generator network G and a discriminator network D operating in an adversarial framework. The generator G synthesizes echo signals from a label vector and noise vector input. Meanwhile, the discriminator D receives both authentic and synthetic signals, aiming to classify them accurately as “real” or “fake” through binary labeling. The networks are optimized adversarially: G strives to generate signals indistinguishable from real data to deceive D, while D is trained to maximize its discrimination accuracy.

**Figure 6:**
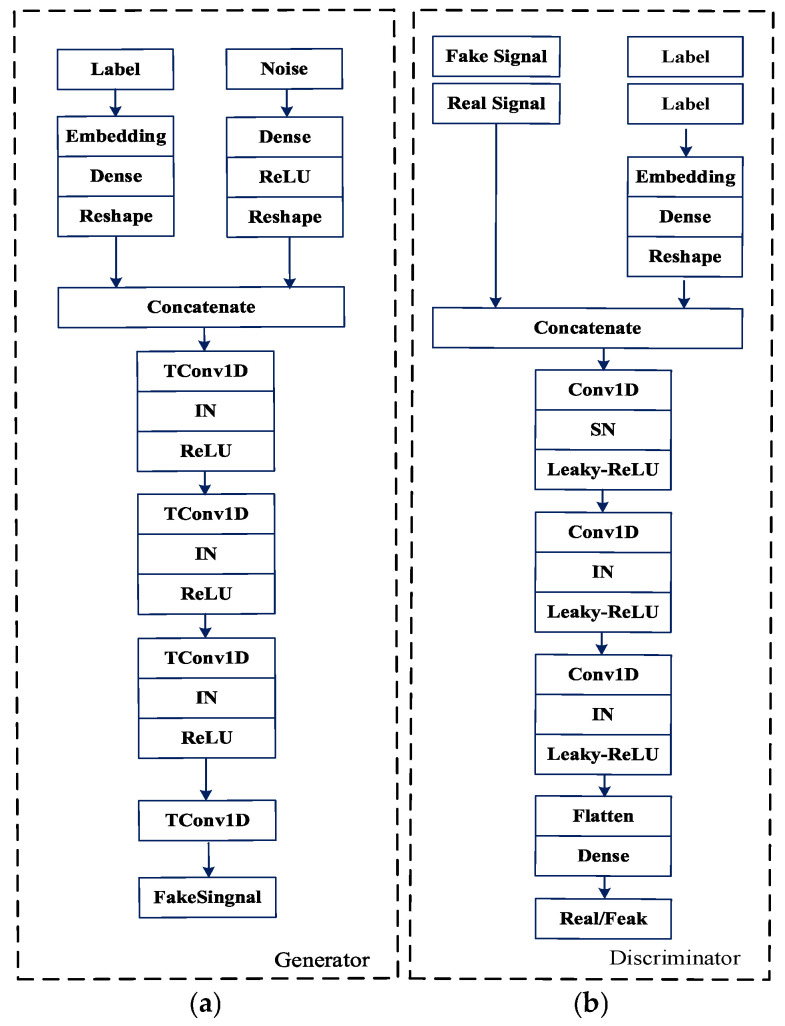
Architecture of the Generator and Discriminator. (**a**) The generator takes a random noise vector as input and outputs a synthetic 1D signal segment. (**b**) The discriminator receives either a real signal from the training set or a generated signal from the generator and predicts its authenticity.

**Figure 7:**
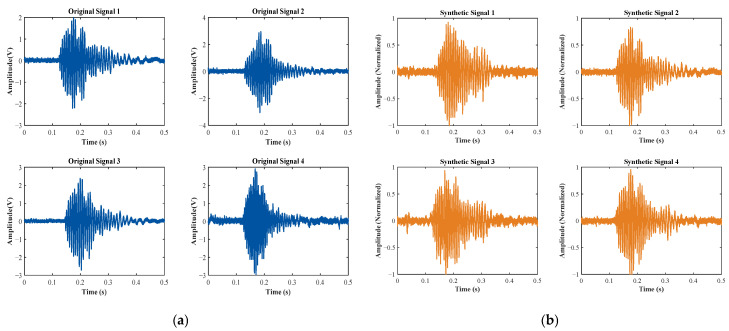
Time-domain waveforms of original and 1D-CGAN-generated water surface echo signals. (**a**) Original; (**b**) synthetic (1D-CGAN-generated).

The model architectures of the generator and discriminator are shown in Figure 6 and Figure 7, respectively, where the generator adopts a “noise-label joint input” encoding strategy that transforms labels into dense vectors via an embedding layer before concatenating them with noise vectors to generate specified counterfeit samples, mapping the joint features to a high-dimensional space through fully connected layers followed by three transposed convolutional layers with ReLU activations implementing feature upsampling to ultimately output category-specific synthetic samples, while the discriminator simultaneously receives both authentic/generated samples and their corresponding labels for feature extraction through four convolutional layers activated by LeakyReLU functions, processes them with global average pooling connected to fully connected layers to execute dual discrimination evaluating sample authenticity and category consistency.

Based on the aforementioned generative adversarial model, 100 sets of synthetic echo signals were generated—50 sets for water surface and 50 sets for grass surface—for subsequent target recognition training.

### 2.4. Quality of Synthetic Echo Signal

The primary objective of the 1D-CGAN is to augment datasets and enhance classifier performance through synthetic data generation. Therefore, evaluating the quality of the generated data constitutes a critical post-training step. To assess this, we compared original echo signals with 1D-CGAN-generated counterparts across three domains: time-domain waveforms, short-time Fourier transform (STFT) analysis, and approximate entropy measurements.

**Figure 8:**
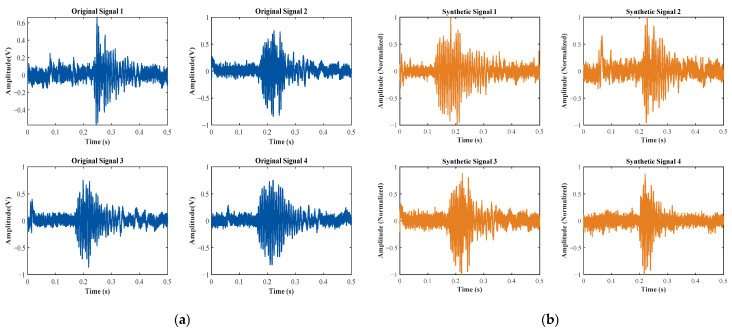
Time-domain waveforms of original and 1D-CGAN-generated grass surface echo signals. (**a**) Original; (**b**) synthetic (1D-CGAN-generated).

Figure 7 and Figure 8 provide a visual comparison between the original time-domain waveforms of water surface and grass surface echo signals and the corresponding synthetic signals produced by the 1D-CGAN. As illustrated in these figures, the generated echo signals exhibit considerable similarity to the original signals in their time-domain characteristics.

**Figure 9:**
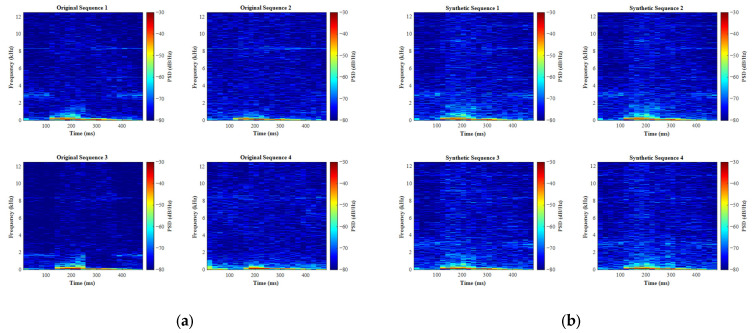
STFT spectrograms of water surface echo signals: original vs. synthetic. (**a**) Original signal; (**b**) 1D-CGAN-generated signal.

**Figure 10:**
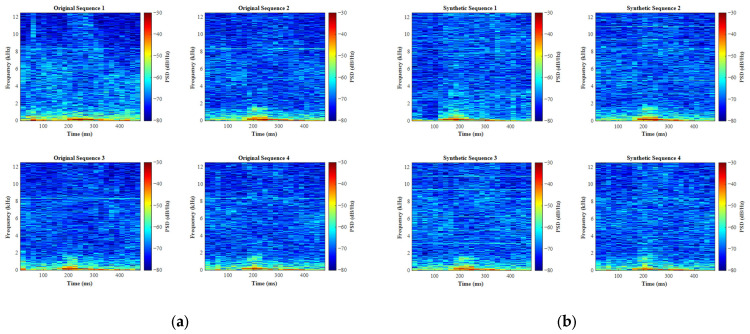
STFT spectrograms of grassland echo signals: original vs. synthetic. (**a**) Original signal; (**b**) 1D-CGAN-generated signal.

Figure 9 and Figure 10 show the STFT spectrograms of water surface and grassland echo signals compared to their synthetic (1D-CGAN-generated) counterparts, respectively. As depicted, the spectrograms of 1D-CGAN-generated signals exhibit visual similarities to the original signals, demonstrating high consistency in their time-frequency distributions.

Approximate Entropy (ApEn), introduced by Steven M. Pincus in 1991, quantifies the complexity and irregularity of time series. We analyze the distributions of approximate entropy to evaluate and contrast the consistency in intrinsic complexity between original echo signals and GAN-generated signals.

When dealing with a known sequence $x\left(i\right)$, the algorithm for calculating the approximate entropy is as follows:

(1) Convert the sequence $x\left(i\right)$ into an m-dimensional vector $\alpha \left(i\right)$:

(3) $$\alpha \left(i\right)=\left[x\left(i\right),x\left(i+1\right),\ldots x\left(i+m-1\right)\right].$$

(2) Define $\;d\left[\alpha \left(i\right),\alpha \left(j\right)\right]$ as the maximum difference between the corresponding elements of $\alpha \left(i\right)$ and $\alpha \left(j\right)$:

(4) $$d\left[\alpha \left(i\right),\alpha \left(j\right)\right]=\underset{k=1,2,\ldots ,m}{max}\left|x\left(i+k-1\right)-x\left(j+k-1\right)\right|.$$

(3) Let *s* denote the similarity tolerance. Then, for every value of i (including i=j) 1≤i≤N−m+1, count the number of vectors that satisfy $d\left[\alpha \left(i\right),\alpha \left(j\right)\right]\leq s$. Let $C_{i}^{m}\left(s\right)$ denote the ratio of this number to the total number of *α*-vectors:

(5) $$C_{i}^{m}\left(s\right)=\frac{1}{N-m-1}num\left\{d\left[\alpha \left(i\right),\alpha \left(j\right)\right]\leq s\right\}.$$

(4) Calculate the Napierian logarithm of $C_{i}^{m}\left(s\right)$ and its mean value $\Phi^{m}\left(s\right)$:

(6) $$\Phi^{m}\left(s\right)=\frac{1}{N-m-1}\sum_{i-1}^{N-m-1}\ln(C_{i}^{m}(s)).$$

(5) Finally, define the approximate entropy of the sequence as follows:

(7) $$E(m,s,N)=\Phi^{m}(s)-\Phi^{m+1}(s),m\geq 2.$$

**Figure 11:**
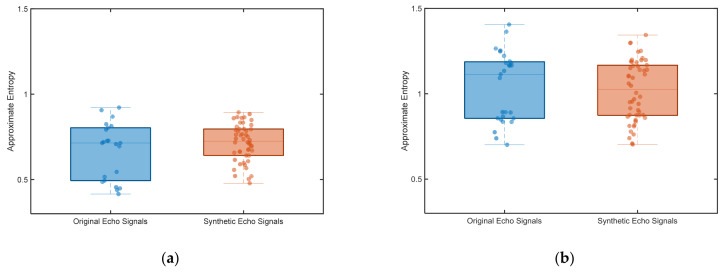
ApEn distribution: original vs. 1D-CGAN-synthetic signals. (**a**) Water surface; (**b**) grass surface.

The approximate entropy values were, respectively, computed for four distinct signal categories: (1) original water surface echo signals, (2) 1D-CGAN-generated water surface signals, (3) original grassland echo signals, and (4) 1D-CGAN-generated grassland signals. Their comparative distributions are presented in Figure 11, where panel (a) analyzes water surface signatures and panel (b) examines grassland signatures.

Analysis reveals substantial agreement between the distribution ranges of approximate entropy for synthesized signals and their authentic counterparts. Although minor disparities in dispersion characteristics are observable, these variations remain within acceptable confidence intervals considering the statistically limited sample size of original signals. From a statistical standpoint, the overall distributional alignment indicates that 1D-CGAN-generated signals effectively capture the essential complexity profiles of authentic environmental signatures.

Through joint analysis of time-domain waveforms, STFT visualizations, and approximate entropy distributions, we conclude that 1D-CGAN-generated signals demonstrate sufficient fidelity to satisfy data augmentation requirements.

### 2.5. Signal Preprocessing

**Figure 12:**
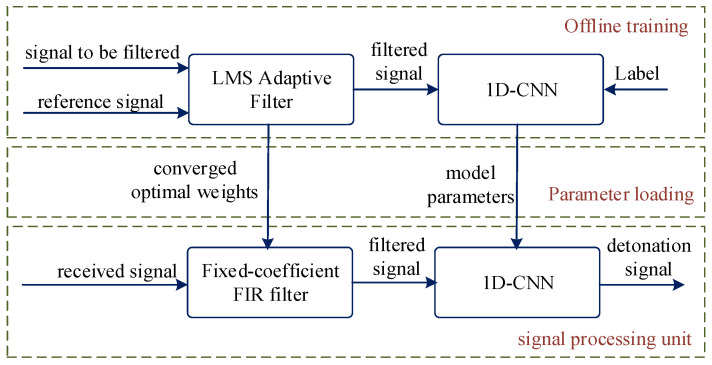
Schematic illustration of offline parameter loading for signal processing unit.

Figure 12 illustrates the training architecture for the UWB fuze signal processing unit: (1) an offline training phase, during which the filter and the 1D-CNN are trained separately to determine their parameters, followed by (2) a deployment phase, during which these parameters are fixed and loaded into the signal processing unit.

For emulating realistic electromagnetic interference scenarios in fuze deployment, the original echo signals were deliberately contaminated with:

- Additive white Gaussian noise μ=0, $\sigma^{2}=0.1$)
- Single-tone interference at 1 GHz (amplitude = 0.2)

**Figure 13:**
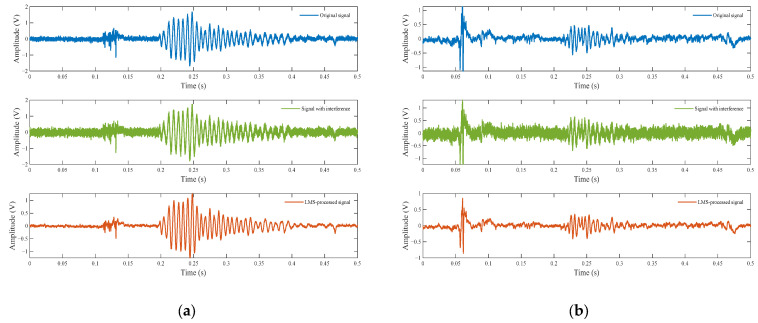
Fixed-Weight LMS filtering performance for interfered echo signals. (**a**) Water surface; (**b**) grass surface.

The echo signal contaminated with noise and interference is input into the LMS adaptive filter, while the low-pass-filtered echo signal serves as the reference signal. With the filter order set to 128, initial weights initialized to zero, and an algorithm step size of $10^{-5}$, a 128-dimensional filter weight vector is obtained through iterative training. This weight vector is then applied to filter other noise- and interference-contaminated echo signals. As evidenced in Figure 13, the offline-trained LMS filter with fixed weights (128-tap) effectively suppresses noise and interference while preserving critical target signatures in noise-contaminated UWB fuze echoes.

**Figure 14:**
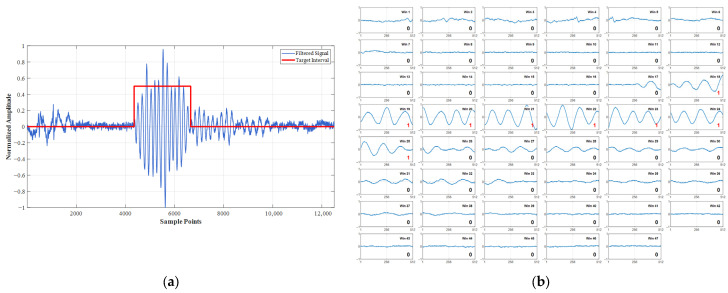
Training Sample Preparation for Sequence Classification Networks. (**a**) Time-domain signal with annotated target region for segmentation; (**b**) 47 × 512-sample windows with label assignments.

Prior to recognition training with the 1D-CNN architecture, all datasets (including both training and test sets) were uniformly segmented using an identical sliding time window procedure (window length: 512 sample points; step size: 256 sample points) to meet fuze real-time processing requirements. This process generated half-overlapping subsequences, each comprising 512 sample points. All resulting subsequences received binary annotation based on target feature presence: Target-present segments were assigned ‘Label 1’, while target-absent segments were assigned ‘Label 0’. The integrated segmentation and annotation methodology is depicted in Figure 14.

### 2.6. Target Recognition Algorithm

Fuze echo signal target recognition is inherently a binary classification problem. The probability distributions from the output layer indicate confidence levels for classifying inputs as target-absent (‘Label 0’) or target-present (‘Label 1’), with the higher-probability label selected as the detonation control signal.

**Figure 15:**
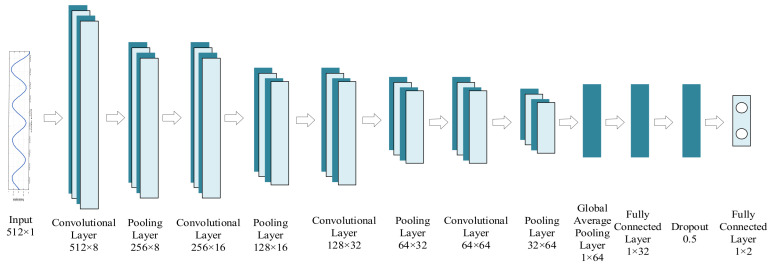
Schematic diagram of the 1D-CNN network model.

To address this task, this paper adopts a 1D-CNN architecture, as illustrated in Figure 15. Its inputs are 512 × 1 time-series subsequences from uniform segmentation. The network contains four consecutive convolutional layers, each paired with pooling operations. Through hierarchical feature extraction, it progressively abstracts signal characteristics from these subsequences. The learned features are flattened, processed by fully connected layers, and utilized for binary classification between target-present (‘Label 1’) and target-absent (‘Label 0’) segments.

To validate the data enhancement effect of the 1D-CGAN, two comparative experimental groups were designed using an identical test set of 10 original signal groups. The first group exclusively employed 40 groups of originally collected signals as the training set. The second group combined 40 groups of original data with 100 groups of 1D-CGAN-generated signals to construct the training set. Both experimental groups encompassed two typical combat surface environments: water and grassland.

All signals underwent identical preprocessing (as described in Section 2.5), which involved adding environmental noise and electromagnetic interference to simulate actual combat conditions, followed by signal enhancement using a fixed-weight vector LMS filter.

Application of this standardized procedure (Figure 14) yielded the following datasets:

(1) Unified test set (10 signal sequences): 470 samples for model validation;
(2) Original training set (40 sequences): 1880 annotated samples;
(3) 1D-CGAN-augmented set (140 sequences): 6580 annotated samples.

Using these prepared samples, the 1D-CNN was trained for target recognition. Following model training and evaluation, significant performance improvements were observed: Recognition accuracy reached 92.34% when trained exclusively on the original-dataset, while accuracy increased substantially to 97.66% when trained on the 1D-CGAN-augmented set.

## 3. Results and Analysis

### 3.1. Comparison with Other Models

**Figure 16:**
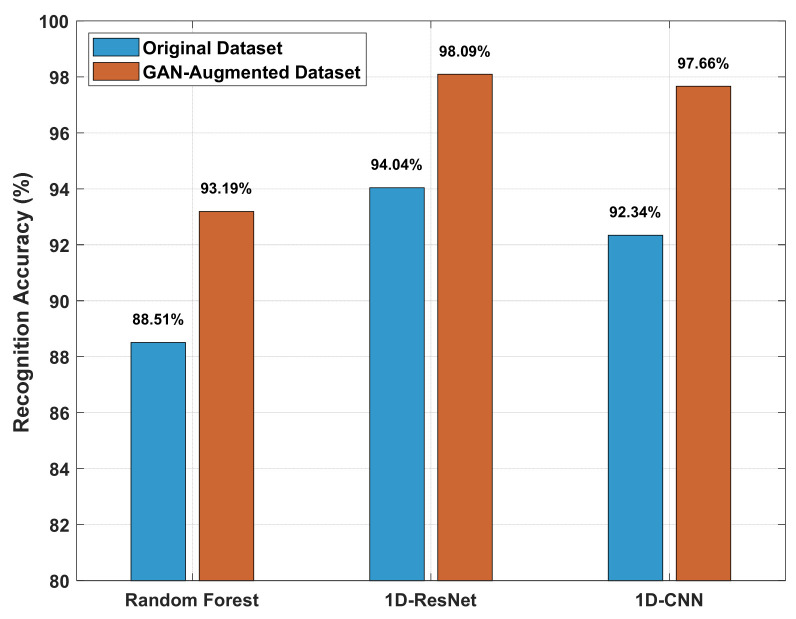
Comparison of Recognition Performance Across Models and Datasets.

To validate the applicability and relative performance of the 1D-CNN architecture within the context of this study, a comparative analysis was conducted against mainstream benchmark models—Random Forest and One-Dimensional Residual Neural Network (1D-ResNet)—under both pre- and post-data augmentation conditions. The Random Forest classifier consisted of 117 decision trees with Gini impurity as the splitting criterion. In contrast, the 1D-ResNet architecture was chosen for its ability to model complex structures within the one-dimensional echo signals; its residual connections facilitate the training of deeper networks and enhance hierarchical feature learning. All models were trained and evaluated on identical datasets under consistent conditions to ensure a fair comparison. The results are summarized in Figure 16.

In terms of recognition accuracy, as depicted in Figure 16, all models exhibited improved performance following 1D-CGAN-based data augmentation. The 1D-CNN architecture adopted in this study achieved competitive accuracy (97.66%), closely approaching the high performance of the 1D-ResNet benchmark (98.09%). While the Random Forest model also showed measurable improvement, it attained a comparatively lower accuracy of 93.19%.

Beyond recognition accuracy, computational efficiency represents another crucial metric for real-time fuze systems. As quantified in Table 1, the 1D-CNN required only 1.38 MFLOPs—approximately seven times fewer computations than the 1D-ResNet architecture (9.65 MFLOPs). This substantial reduction in computational overhead demonstrates that while 1D-ResNet achieves marginally higher accuracy, it does so at a significant computational cost.

Due to fundamental differences in architectural operation—floating-point operations (CNN/ResNet) versus logical branching (Random Forest)—direct computational comparison with Random Forest is not analytically meaningful. Nevertheless, its lower recognition accuracy and inherent limitations in hardware acceleration further highlight the practical advantages of the 1D-CNN architecture.

Collectively, these results demonstrate that the 1D-CNN achieves a favorable balance between recognition accuracy and computational efficiency. Considering the real-time requirements of the fuze system, the 1D-CNN maintains acceptable recognition accuracy while operating at a relatively low computational cost, making it particularly suitable for resource-constrained applications where both performance and operational practicality are critical.

### 3.2. System-Level Performance Evaluation

Building upon the segment-level recognition accuracy of 97.66% achieved by the 1D-CNN model trained on the 1D-CGAN-augmented dataset, as discussed in the previous section, it is imperative to emphasize that this metric pertains solely to isolated segment-level classification. Each complete echo signal was partitioned into 47 segments during preprocessing to facilitate deep feature learning. To accurately assess the performance of the signal processing model on complete echo signals within the context of fuze application, the following system-level metrics are established according to the mission-critical demands of the fuze system:

(1) Miss detection: All target-present segments in a complete signal are misclassified as ‘Label 0’.
(2) False alarm: Any target-absent segment misclassified as ‘Label 1’ prior to the first true target segment.
(3) Fuze detonation position: The system triggers the fuze immediately at the temporal position corresponding to the first segment classified as ‘Label 1’. The classification of all subsequent segments is irrelevant once the detonation signal is issued.

**Figure 17:**
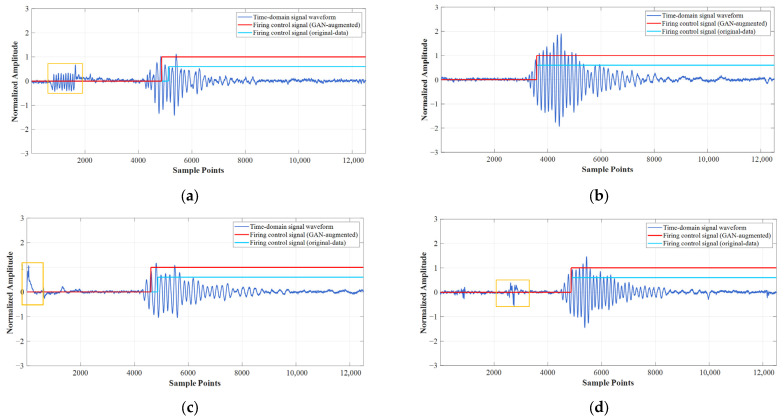
Representative Test Data Subset. (**a**) Grass Surface Identification Scenario: Case 1; (**b**) Water Surface Identification Scenario: Case 1; (**c**) Grass Surface Identification Scenario: Case 2; (**d**) Water Surface Identification Scenario: Case 2.

As shown in Figure 17, which illustrates representative test results of reconstructed full-echo signal identification. The red firing control signal is triggered when the 1D-CNN model trained with 1D-CGAN-augmented data detects the first signal segment classified as ‘Label 1’. The cyan firing control signal is triggered when the 1D-CNN model trained on original data detects the first segment classified as ‘Label 1’.

Although pre-filtering processing was applied using LMS, residual interference clutter remains preceding the echo signal in some cases, as shown in the yellow boxes in Figure 17a,c,d.

In traditional threshold detection methods, a threshold is typically preset based on engineering experience. During signal processing, the amplitude of the echo signal is compared against this preset threshold: if the signal amplitude exceeds the threshold, a target is deemed present, and a detonation signal is output; otherwise, no target is considered detected. This approach introduces an inherent dilemma: setting the threshold too high results in missed detections when the echo signal amplitude is weak, while setting it too low leads to false alarms when interference signals exceed the threshold. Therefore, if the residual interference clutter remaining after LMS filtering exceeds this threshold, it may trigger a detonation signal, thereby causing a false alarm.

In contrast, on our test dataset, both 1D-CNN models—whether augmented with 1D-CGAN or trained on original data—achieved 0% false alarm and 0% missed detection rates. This is primarily attributed to two factors: (1) the high per-segment accuracy (97.66% for the 1D-CNN model trained with 1D-CGAN-augmented data and 92.34% for the 1D-CNN trained on original data), which made the probability of missing all target segments across all segments of a complete echo signal exceedingly low; and (2) through learning target-related features, the 1D-CNN model can accurately label clutter and interference signals as ‘Label 0’.

As clearly evidenced by the comparative results in Figure 17a,c, the 1D-CGAN-augmented model demonstrates significantly superior reliability in initial target segment detection—a critical capability for precise detonation control. Whereas the baseline model (trained on original data) exhibited first-segment misidentification in four test cases, resulting in a delayed firing signal, the augmented model misidentified the initial target segment in only a single instance. The remaining errors observed in the augmented model were confined to the tail section of the signal, which bears no impact on detonation triggering since the firing decision depends exclusively on the earliest confident target detection. This marked improvement in first-segment recognition reliability directly translates to enhanced precision in detonation positioning, underscoring the tactical value of 1D-CGAN-based augmentation for fuze applications where accurate and timely target initiation is paramount.

## 4. Conclusions

To address the reliability limitations of UWB fuzes in complex electromagnetic environments while meeting real-time requirements, this paper proposes a novel signal processing framework for UWB fuzes. Simulation results validate its effectiveness with the following key conclusions:

First, under deliberate jamming conditions, the proposed UWB fuze signal processing framework achieved 0% false alarm rate and 0% miss detection rate in test scenarios.

Second, 1D-CGAN data augmentation elevated the 1D-CNN recognition success rate to 97.66%, representing a 5.32% increase over the non-augmented baseline that substantially enhances target identification model reliability. Finally, 1D-CGAN data augmentation resolved the low recognition rate for initial target signal windows observed in the baseline 1D-CNN model trained on raw data, directly enhancing UWB fuze detonation position control precision.

This study successfully implements 1D-CGAN and 1D-CNN in UWB fuze signal processing to resolve critical limitations of traditional energy-threshold detection methods. Specifically, it addresses reliability degradation under interference conditions while overcoming neural network training deficiencies caused by scarce echo signal data. Furthermore, through comparative analysis with other mainstream models— Random Forest and ResNet—the 1D-CNN is validated as a well-suited architecture for the real-time processing constraints of fuze applications, achieving an effective balance between recognition accuracy and computational overhead. This advancement significantly enhances detonation position control for UWB fuzes operating in complex electromagnetic environments.

## Figures and Tables

**Table 1:** Comparison of Computational Complexity (FLOPs) Across Models.

Model	Flops
1D-ResNet	9,654,508
1D-CNN	1,375,036

## Data Availability

The original contributions presented in the study are included in the article, further inquiries can be directed to the corresponding author.
